# Effect of Fluoride-Modified Titanium Surface on Early Adhesion of Irradiated Osteoblasts

**DOI:** 10.1155/2015/219752

**Published:** 2015-07-22

**Authors:** Jun Yuan Li, Li Wu Zheng, Li Ma, Dora Lai Wan Kwong, Lim Kwong Cheung, Edmond Ho Nang Pow

**Affiliations:** ^1^Oral Rehabilitation, Faculty of Dentistry, The University of Hong Kong, Pokfulam, Hong Kong; ^2^Oral Diagnosis and Polyclinics, Faculty of Dentistry, The University of Hong Kong, Pokfulam, Hong Kong; ^3^Department of Clinical Oncology, Li Ka Shing Faculty of Medicine, The University of Hong Kong, Pokfulam, Hong Kong; ^4^Oral and Maxillofacial Surgery, Faculty of Dentistry, The University of Hong Kong, Pokfulam, Hong Kong

## Abstract

*Objective*. The present study aimed to investigate the effect of fluoride-modified titanium surface on adhesion of irradiated osteoblasts. *Materials and Methods*. Fluoride-modified surface was obtained and the morphology, roughness, and chemical composition of the surface were evaluated by scanning electron microscopy, atomic force microscopy, and X-ray photoelectron spectroscopy, respectively. The adhesion of irradiated osteoblast-like cells, in terms of number, area, and fluorescence intensity on the titanium surface, was evaluated using immunofluorescence staining. *Results*. Numerous nanosize pits were seen only in the F-TiO surface. The pits were more remarkable and uniform on F-TiO surface than on TiO surface; however, the amplitude of peaks and bottoms on F-TiO surface appeared to be smaller than on TiO surface. The Sa value and Sdr percentage of TiO surfaces were significantly higher than those of F-TiO surface. The concentrations of main elements such as titanium, oxygen, and carbon were similar on both surfaces. The number of irradiated osteoblasts adhered on the control surface was larger than on fluoride-modified surface. Meanwhile, the cells on the fluoride-modified surface formed more actin filaments. *Conclusions*. The fluoride-modified titanium surface alters the adhesion of irradiated osteoblasts. Further studies are needed to investigate the proliferation, differentiation, maturation, gene expression, and cytokine production of irradiated osteoblasts on fluoride-modified titanium surface.

## 1. Introduction

Radiotherapy is known to compromise bone quality via endothelial cell death, microvascular thrombosis, reduced number of osteoblasts, and lowered oxygen tension [1]. The survival of implants placed in irradiated bone is less predictable and its failure is largely due to the poor bone formation around implants [2].

Cell attachment is crucially important for biomaterial-bone integration. It is the initial process of bone healing and remodeling, which significantly influences the subsequent cell differentiation and cell mineralization [3, 4]. Cell attachment can be affected by cell type, cell activity, and expression of adhesion protein, as well as the topography, chemistry, roughness, energy, and surface hydrophobicity of implant surface [5, 6].

Osseointegration of dental implants involves the recruitment of multipotent mesenchymal stem cells and the differentiation of these cells into osteoblasts. The promotion of osteoblast attachment and differentiation has been evaluated on various implant surfaces using a variety of* in vitro* and* in vivo* experimental models. It has been reported that fluoride can increase osteoblast number, promote bone formation rate, and enhance serum concentration of alkaline phosphatase (ALP) [7]. Studies showed that fluoride-modified titanium surface increased osteoblast proliferation, induced high level expression of osteogenic markers, promoted bone mineralization, and enhanced bone formation in cell culture [8, 9] and animal experiment [10, 11].

However, so far to our knowledge no studies have reported the influence of fluoride-modified titanium surface on cell attachment after radiation. To investigate the hypothesis that fluoride-modified implant may promote adhesion of irradiated cells, the adhesion of irradiated osteoblast-like cells on fluoride-modified titanium surface was evaluated.

## 2. Materials and Methods

### 2.1. Test Materials

Two types of titanium disks with different surface treatments were used. The diameter and thickness of the disks are 6.25 mm and 2.0 mm, respectively. The control disks (TiO) were prepared by grade II titanium with dioxide grit blasted surface according to TiOblast manufacturing procedure (DENTSPLY Implants, Mölndal, Sweden). The test disks (F-TiO) were prepared on the basis of control disks followed by electrochemical etching in hydrofluoric (HF) acid solution according to OsseoSpeed manufacturing procedure (DENTSPLY Implants, Mölndal, Sweden). Organic contaminants were removed from the surface of the disks. Beta-irradiation was used for sterilization before sealing the disks in the containers.

### 2.2. Scanning Electron Microscopy (SEM)

The samples were directly mounted on aluminum stubs without coating for SEM analysis (Hitachi S-4800, Hitachi High-Technologies Corp., Tokyo, Japan). Vacuum pressure was maintained during scanning. The surfaces were observed at 6000x and 18000x magnifications.

### 2.3. Atomic Force Microscopy (AFM)

AFM (Veeco Instruments, Santa Barbara, CA, USA) was used to assess morphology of micro- and nanolevel surface and measure surface roughness quantitatively. Standard silicon cantilever tips (OMCL-AC160TS, Olympus, Tokyo, Japan) with resonant frequency of 300 kHz and spring constant of 42 N/m were used. The scanning was performed at room temperature with a scanning size of 1 *μ*m × 1 *μ*m. Six random measurements were taken for each sample. Average height deviation from a mean plane within the measuring area (Sa), maximum difference of peak and concaved bottom (St), and additional surface area contributed by the roughness compared with the area of flattened surface (Sdr) were calculated by AFM package software.

### 2.4. X-Ray Photoelectron Spectroscopy (XPS)

The surface chemical composition was investigated by XPS (ESCALAB 250, Thermo Fisher Scientific, Florida, US) with an Al K*α* monochromatic X-ray source and a source power of 15 kV, 150 W. Vacuum pressure of 2 × 10^−9^ mbar was maintained during XPS scanning. Three spots were randomly selected on each sample with atomic concentration (%) of main elements measured.

### 2.5. Cell Culture

Human osteoblast-like SaOS-2 cells (catalog number HTB-85, ATCC, Manassas, VA, USA) were cultured in McCoy's 5A medium (Sigma Aldrich, UK) supplemented with 10% fetal calf serum (FCS, Sigma Aldrich, UK), 100 U/mL penicillin, and 0.1 mg/mL streptomycin and incubated at 37°C with 5% CO_2_. The culture medium was changed every 2 days.

### 2.6. Radiotherapy

**Figure 1:**
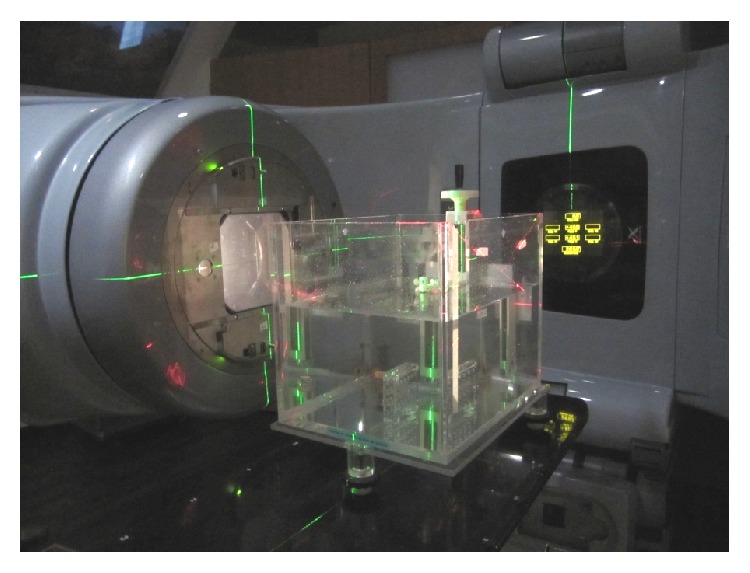
Electron radiation of cells in the water tank.

The cells were radiated with a linear accelerator radiotherapeutic machine (Varian, Palo Alto, CA, USA) in Department of Clinical Oncology, Queen Mary Hospital, The University of Hong Kong. Cells were harvested with density of 2 × 10^4^ cell/mL, collected in 1 mL tubes, and stored in the icebox. In order to ensure uniform radiation distribution, the tubes containing cells were placed in the water tank, and electron radiation rays went through the center of tubes (Figure 1). The dose rate was 400 cgy/min. The cells received 0 Gy, 2 Gy, 4 Gy, 6 Gy, 8 Gy, or 10 Gy radiation respectively.

### 2.7. Immunofluorescence Staining

After irradiation, 40 *μ*L cell suspension was seeded onto the disk surface to form a bead. Four hours later, 2 mL culture medium was carefully added until the whole disk was immersed. Twenty-four hours after seeding, cells were fixed with 4% paraformaldehyde (Sigma Aldrich, Taufkirchen, Germany) in PBS for 10 min at room temperature. The disks were rinsed three times in PBS.

4,6-Diamidino-2-phenylindole (DAPI) was added on the disk to stain the cell nuclei blue specifically. Alexa Fluor phalloidin-555 (Invitrogen, CA, USA) was used to stain F-actin filaments according to the method previously described by Muthukumaran et al. [12]. Briefly, the membrane of cells was permeated by PBS containing 0.1% Triton X-100 and blocked with 1% bovine serum albumin in PBS. The cells were treated with 10 *μ*g/mL phalloidin solution to label the actin cytoskeleton. Images of two fluorescent channels (DAPI and phalloidin) were viewed under confocal laser scanning microscope (Olympus FluoView FV1000, Olympus, Tokyo, Japan). Overlaying of the images was performed with package software (Olympus FluoView software, Tokyo, Japan).

**Figure 2:**
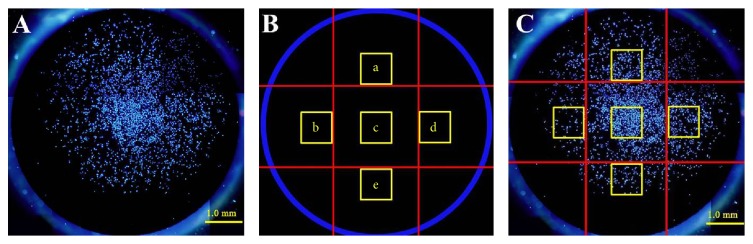
SaOS-2 osteoblasts attached on titanium disks under fluorescence microscope. (A) SaOS-2 attached on Ti disk which are stained by DAPI. The number of cells on the whole disks is measured. (B) The Ti disk is divided into nine areas. Five yellow squares (a–e) are selected. (C) Cell area and fluorescence intensity of cells in five yellow squares were evaluated.

The number of cells on the whole disks was measured by Image J software (Figure 2(A)). After measuring cells number, the titanium disk was divided into nine areas. The mean cell area and cell fluorescence intensity in five areas (Figures 2(B) and 2(C)) were evaluated as described in previous studies [13, 14].

### 2.8. Statistical Analysis

The data was analyzed using version 19.0 of Statistical Package of Social Sciences software (SPSS Inc., Chicago, IL, USA). The preliminary analysis showed that the data did not meet the criteria for parametric tests; therefore, nonparametric analyses were used. Differences between the radiation group and the nonradiation group under the same dosage were tested by Mann-Whitney tests, while the values among different doses were analyzed by Kruskal-Wallis test and further post hoc comparisons were performed if the significance was detected. Differences were considered significant at *P* ≤ 0.05.

## 3. Results

### 3.1. Surface Morphology

**Figure 3:**
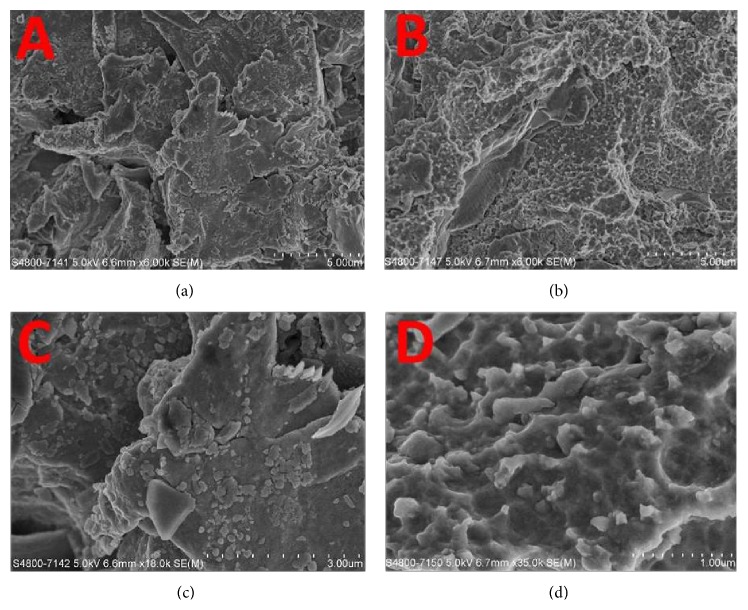
Scanning electron microscopic images of TiO surface and F-TiO surface. (a) TiO surface with magnification of 6000; (b) F-TiO surface with magnification of 6000; (c) TiO surface with magnification of 18000; (d) F-TiO surface with magnification of 18000.

SEM examination found that the TiO surface and F-TiO surface showed isotropic properties (Figure 3). At low magnification (Figures 3(a) and 3(b)), TiO surface and F-TiO surface had no significant difference. At high magnification (Figures 3(c) and 3(d)), a distinct topography with numerous nanosize pits was seen only in the F-TiO surface.

**Figure 4:**
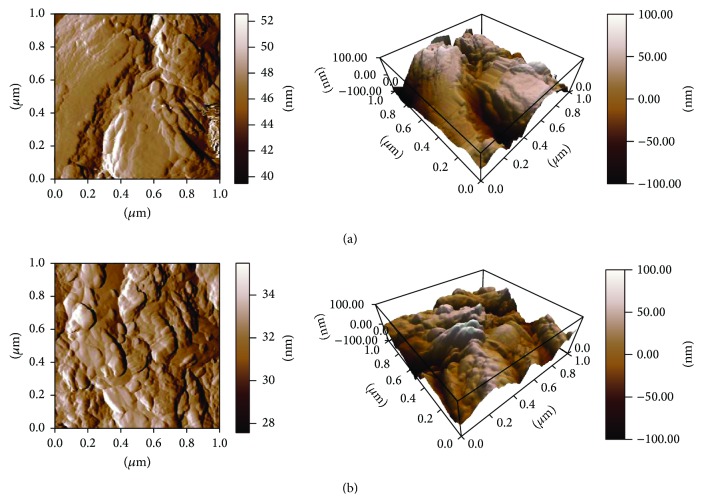
TiO surface and F-TiO surface under atomic force microscopy with the magnification of 1 *μ*m × 1 *μ*m. (a) TiO surface; (b) F-TiO surface.

AFM analysis demonstrated that the pits were more remarkable and uniform on F-TiO surface than on TiO surface (Figure 4). However, the amplitude of peaks and bottoms on F-TiO surface appeared to be smaller than on TiO surface.

### 3.2. Surface Roughness

AFM analysis showed that the Sa value and Sdr percentage of TiO surfaces were significantly higher than those of F-TiO surface (Table 1).

### 3.3. Surface Composition

XPS examination found that the concentrations of main elements such as titanium, oxygen, and carbon were similar on both surfaces (Table 2). However, trace amounts of fluorine (1.35%) and nitrogen (1.33%) were found on the F-TiO surfaces, which were not detected on the TiO surfaces.

### 3.4. Cell Morphology

**Figure 5:**
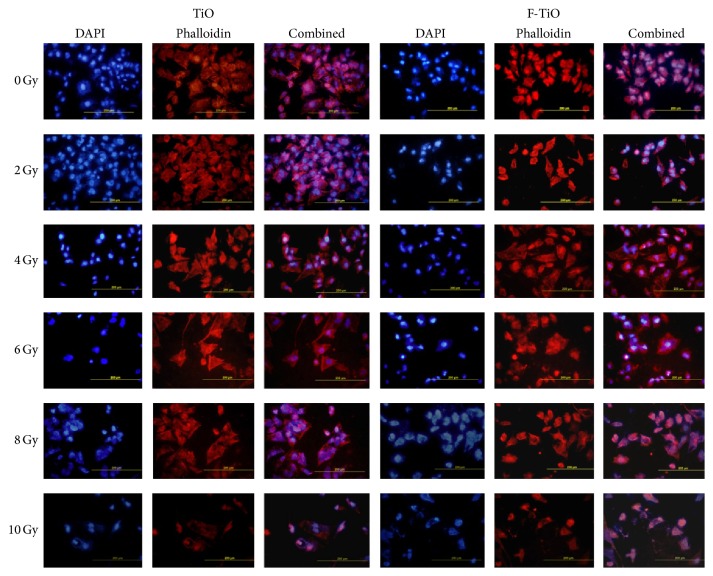
SaOS-2 osteoblasts attached on titanium disks under fluorescence microscope. The nuclei are stained blue by DAPI, and F-actin cytoskeletons are stained red by phalloidin.

Figure 5 showed typical fluorescence stained cells under different radiation dosages. The seeded cells formed polygonal shape on the titanium surface. Cells exhibited processes connecting neighboring cells. With increasing dosage of radiation, stimulated cell division accompanied by increase in nuclei size but reduction in cytoplasm was observed. In addition, the border of the attached cells appeared to be less ruffled. Cells that received high dose of radiation showed signs of apoptosis, such as irregular nuclei shape, condensed nuclei, and loss of cellular processes. Comparing the two surfaces, the cells on TiO surfaces were larger and flatter than those on F-TiO surfaces under the same radiation dosage (Figure 5).

### 3.5. Cells Number

Radiation dose had significant effect on cells number for both TiO group (*P* = 0.016) and F-TiO group (*P* = 0.018) (Table 3). On the TiO surface, the number of attached cells that received 10 Gy radiation was significantly lower than that of cells that received 0 Gy (*P* = 0.001) and 2 Gy (*P* = 0.009) radiation. Significantly fewer cells were also found on TiO surfaces after 6 Gy (*P* = 0.039) and 8 Gy radiation (*P* = 0.018) when compared to those after 0 Gy radiation. On the F-TiO surface, the number of attached cells under 10 Gy radiation was significantly lower than that under 0 Gy (*P* = 0.002), 2 Gy (*P* = 0.012), and 4 Gy (*P* = 0.032) radiation. In addition, significantly fewer cells were attached after 8 Gy radiation when compared to those after 0 Gy radiation (*P* = 0.014). When the two surfaces were compared, the number of osteoblasts on F-TiO surface was marginally significantly lower than those on TiO surface after 0 Gy (*P* = 0.05), 2 Gy (*P* = 0.05), 8 Gy (*P* = 0.05), and 10 Gy (*P* = 0.05) radiation.

### 3.6. Cell Area

With increasing dosage of radiation, significant difference of cell area was detected neither on TiO surface (*P* = 0.150) nor on F-TiO surface (*P* = 0.155) (Table 4). For the cells that received 2 Gy radiation, the cell area of osteoblasts on TiO surface was marginally significantly larger than that on F-TiO surface (*P* = 0.05). However, for the cells that received 6 Gy radiation, the cell area of osteoblasts on TiO surface was marginally significantly less than that on F-TiO surface (*P* = 0.05).

### 3.7. Fluorescence Intensity

Radiation dosage had significant impact on fluorescence intensity of cells attached on both TiO surface (*P* = 0.017) and F-TiO surface (*P* = 0.009) (Table 5). For cells on TiO surface, fluorescence intensity of cells that received 10 Gy radiation was significantly lower than those that received 2 Gy radiation (*P* = 0.012). Cells that received 4 Gy (*P* = 0.032), 8 Gy (*P* = 0.027), and 10 Gy (*P* = 0.001) radiation had significantly lower fluorescence intensity than those that received 0 Gy. For cells on F-TiO surface, fluorescence intensity of cells that received 10 Gy radiation was significantly lower than those that received 0 Gy (*P* = 0.001) and 2 Gy (*P* = 0.012) radiation. Moreover, fluorescence intensity of cells that received 8 Gy radiation was significantly lower than those that received 0 Gy (*P* = 0.005) and 2 Gy (*P* = 0.032) radiation. Cells that received 6 Gy radiation also had significantly lower fluorescence intensity than those that received 0 Gy (*P* = 0.039).

Comparing the two surfaces, the fluorescence intensity of cells on F-TiO surface was marginally significantly higher than cells on TiO surface after receiving 0 Gy (*P* = 0.05), 2 Gy (*P* = 0.05), 4 Gy (*P* = 0.05), and 10 Gy (*P* = 0.05) radiation.

## 4. Discussion

Radiation causes bone tissue hypoxia, hypocellularity, and hypovascularity, which leads to endarteritis obliterans, periosteal fibrosis, and disturbance to osteogenic cells. The gradual loss of bone cells after radiotherapy will consequently influence the bone remodeling process [15] and that might be one of the reasons why dental implants placed in irradiated bone fail [16].

Osseointegration is determined by the recruitment and migration of osteogenic cells towards the implant surface [17]. Surface modification of titanium implant may promote the bone healing process. Numerous attempts to modify titanium implant surfaces were made to attract cells to the implant surface and improve cell behavior on the surface. Modified surface may improve cytoskeletal structure and enhance actin microfilaments to attach to the substrate, thus promoting cell adhesion and cell migration [18]. Fluoride modification of titanium dental implants has potential for promoting bone response [19], reducing the healing time [20], improving the bone-to-implant contact [21], and stimulating osteoblast gene expression at the implant surface [10]. The present study investigated the adhesion of irradiated osteoblasts to fluoride-modified titanium surface and quantitatively analyzed the cells number, cell area, and immunofluorescent intensity.

In the current study, the coverage of radiation dosage from 0 to 10 Gy was broad enough to detect changes of cell responses [22]. Instead of delivery of radiation to the cells seeded on titanium discs, cells were irradiated in suspension before they were seeded which could avoid scattering of radiation from titanium and ensure more homogeneous and accurate dose of radiation [22, 23]. SaOS-2 cells were selected because they are derived from human, which are homogenous, immortalized and have similar reactions including cytokine and growth factor expressions to titanium substrate like normal human osteoblast cells [24, 25]. Cells number was directly measured on the whole titanium disk to avoid any loss of cells during trypsinizing and collecting of the cells [26]. Fluorescence microscopy was used to facilitate the assessment of cell morphology, cell area, and cell fluorescent intensity at the same time with minimal disturbance to the cells.

The atomic concentration of fluoride on titanium surface was 0.45% which is within the range (0.3%–1%) of other studies in the same field [8, 27]. The topographic changes of blasted titanium surface following fluoride treatment are inconclusive in the literature. Some studies showed that the surface roughness was increased [28], while others revealed reduced [29] or no significant change in surface roughness after hydrofluoric acid treatment [30]. The results of the present study showed that the roughness of fluoride-modified surface was significantly lower than the nonmodified surface. The discrepancy might be due to the variation in measuring equipment as many studies used optical profilometer instead of AFM. In addition, the setting of AFM parameters could also affect the results [31].

Under the same dosage of radiation, the osteoblasts on TiO surface were more than on F-TiO surface, while the actin intensity of cells on F-TiO was higher than on TiO surface. This indicated that the irradiated osteoblasts were prone to adhere onto the TiO surface; on the other hand, the cells on the F-TiO surface formed more actin filaments. It is still unclear why the actin filaments of osteoblasts were enhanced on fluoride-modified surface while the number of cells did not increase correspondingly. Previous studies also showed that fluoride modification of titanium surface did not influence the number of attached osteoblasts but improved cell differentiation [8, 9]. One possible explanation is that the chemical composition and morphology of the surface may change the signal translation in the cell, which in turn alters the pattern of cell adhesion and cell spread. Increased actin cytoskeleton often implies an increase of cell focal contact to the substrate [32]. Actins can mediate various transmembrane signal transductions and activate the cascade of osteoblast proliferation, differentiation, and mineralization [33]. Therefore, it is reasonable to speculate that F-TiO might have the potential to promote the proliferation and differentiation of osteoblasts, which should be confirmed in the future studies.

Regarding the effect of radiation on the osteoblast cells, a dose-dependent effect of radiation on cells number and cell fluorescent intensity was found. With the increase of radiation dosage, the cells number and cell fluorescent intensity were reduced. The number of irradiated osteoblasts attached on the fluoride-modified surface was significantly lower than that on the control surface with the radiation dosage of 0 Gy, 2 Gy, 8 Gy, and 10 Gy. The fluorescent intensity of cells on F-TiO surface was higher than on TiO surface under each radiation dosage and a significant difference was detected at 0 Gy, 2 Gy, 4 Gy, and 10 Gy. However, cell area did not follow a certain pattern as radiation caused reversible or irreversible cell death, which consequently led to irregular cell morphology and uncertain cell area [34]. To our knowledge, no previous study intensively investigated the adhesion, particularly cell area and actin filament intensity, of irradiated osteoblasts on titanium and fluoride-modified surface. The reaction of irradiated osteoblasts to fluoride-modified titanium surface might not be the same as the other reported titanium surfaces [35]. Time might also be an important factor to be considered when explaining the results. Fluoride may require a certain exposure period (around 7 days) to attract higher number of osteoblasts [36]. Meanwhile, the influence of radiation on osteoblasts might not be immediate and start at 24–48 hours after radiation [37]. In order to better understand the effects of radiation on cell behavior on fluoride-modified titanium surface, further experiments are certainly needed to determine the optimal time for seeding the irradiated osteoblasts and measuring the cell response. The proliferation, differentiation and maturation of irradiated osteoblasts on fluoride-modified titanium surface should also be investigated.

## 5. Conclusions

The present study showed that the fluoride-modified surface did affect the adhesion of irradiated osteoblasts. The number of irradiated osteoblasts adhered on the control surface was larger than on fluoride-modified surface. Meanwhile, the cells on the fluoride-modified surface formed more actin filaments. Further studies are needed to investigate the proliferation, differentiation, and maturation of irradiated osteoblasts on fluoride-modified titanium surface.

## Figures and Tables

**Table 1:** Surface roughness (mean ± S.D.) from AFM analysis.

Group	Sa (nm)	St (nm)	Sdr (%)
TiO	42.60 ± 7.70	226.71 ± 48.88	36.51 ± 7.76
F-TiO	28.34 ± 7.24	186.49 ± 66.12	19.54 ± 6.40
*P* value	0.014^*∗*^	0.306	0.005^*∗*^

^*∗*^ *P* < 0.05, significantly different.

**Table 2:** Atomic concentration (%) of samples measured by XPS.

Group	Ti	O	C	K	F	N
TiO	20.36	55.66	23.62	0.36	—	—
F-TiO	14.31	43.15	39.09	0.77	0.45	1.33

**Table 3:** Cells number of SaOS-2 attached on titanium disks under different dosage of radiation.

Radiation dose	Ti disk	*N*	Mean	Standard deviation	Min	Max	Median	95% confidence interval		*P* value
								Lower	Upper	
0 Gy	TiO	3	2751.7	328.8	2453.0	3104.0	2698.0	1934.9	3568.5	0.050^*∗*^
	F-TiO	3	2138.7	286.0	1853.0	2425.0	2138.0	1428.2	2849.1	

2 Gy	TiO	3	2331.0	207.5	2122.0	2537.0	2334.0	1815.5	2846.5	0.050^*∗*^
	F-TiO	3	1907.0	195.0	1713.0	2103.0	1905.0	1422.6	2391.4	

4 Gy	TiO	3	2008.7	198.5	1796.0	2189.0	2041.0	1515.6	2501.7	0.513
	F-TiO	3	1822.0	351.4	1580.0	2225.0	1661.0	949.2	2694.8	

6 Gy	TiO	3	1907.0	198.5	1689.0	2077.0	1956.0	1414.2	2400.5	0.127
	F-TiO	3	1585.0	262.0	1323.0	1847.0	1585.0	934.2	2235.8	

8 Gy	TiO	3	1841.3	153.3	1675.0	1977.0	1872.0	1460.5	2222.2	0.050^*∗*^
	F-TiO	3	1329.0	174.9	1173.0	1518.0	1296.0	894.6	1763.4	

10 Gy	TiO	3	1586.7	198.9	1360.0	1732.0	1668.0	1092.6	2080.7	0.050^*∗*^
	F-TiO	3	1108.7	182.6	923.0	1288.0	1115.0	655.1	1562.2	

^*∗*^ *P* = 0.05, marginally significantly different.

**Table 4:** Cell area of SaOS-2 attached on titanium disks under different dosage of radiation.

Radiation dose	Ti disk	*N*	Mean	Standard deviation	Min	Max	Median	95% confidence interval		*P* value
								Lower	Upper	
0 Gy	TiO	3	1033.2	149.9	878.9	1178.2	1042.5	660.9	1405.5	0.513
	F-TiO	3	1208.2	302.9	996.6	1555.2	1072.8	455.7	1960.7	

2 Gy	TiO	3	1100.8	235.2	847.9	1313.0	1141.5	516.5	1685.0	0.050^*∗*^
	F-TiO	3	706.7	80.2	618.8	775.6	725.8	507.6	905.8	

4 Gy	TiO	3	1031.3	249.0	812.4	1302.1	979.2	412.7	1649.8	0.513
	F-TiO	3	1083.0	78.9	1000.0	1156.9	1092.4	887.0	1279.1	

6 Gy	TiO	3	879.3	221.6	627.4	1044.4	966.0	328.8	1429.8	0.050^*∗*^
	F-TiO	3	1243.1	117.7	1131.9	1366.3	1230.9	950.7	1535.4	

8 Gy	TiO	3	1600.3	285.7	1351.8	1912.5	1536.7	890.6	2310.0	0.275
	F-TiO	3	1313.8	407.7	990.0	1771.6	1180.0	301.1	2326.5	

10 Gy	TiO	3	930.7	247.7	702.2	1194.0	895.9	315.3	1546.1	0.127
	F-TiO	3	1275.6	322.9	967.2	1611.3	1248.2	473.4	2077.8	

^*∗*^ *P* = 0.05, marginally significantly different.

**Table 5:** Fluorescence intensity of SaOS-2 attached on titanium disks.

Radiation dose	Ti disk	*N*	Mean	Standard deviation	Min	Max	Median	95% confidence interval		*P* value
								Lower	Upper	
0 Gy	TiO	3	12.44	1.18	11.46	13.75	12.11	9.51	15.37	0.050^*∗*^
	F-TiO	3	16.69	2.19	15.43	19.22	15.43	11.26	22.12	

2 Gy	TiO	3	5.38	1.30	3.89	6.31	5.94	2.14	8.61	0.050^*∗*^
	F-TiO	3	10.04	3.20	7.85	13.72	8.57	2.09	18.00	

4 Gy	TiO	3	3.09	1.44	1.59	4.46	3.20	.049	6.66	0.050^*∗*^
	F-TiO	3	5.69	0.73	5.16	6.52	5.39	3.88	7.50	

6 Gy	TiO	3	3.67	0.52	3.10	4.13	3.78	2.37	4.97	0.275
	F-TiO	3	4.30	1.28	3.11	5.66	4.14	1.12	7.48	

8 Gy	TiO	3	3.39	0.41	2.93	3.74	3.49	2.36	4.41	0.827
	F-TiO	3	3.28	0.42	3.02	3.76	3.05	2.24	4.31	

10 Gy	TiO	3	1.37	0.27	1.15	1.68	1.28	0.69	2.05	0.050^*∗*^
	F-TiO	3	2.97	0.43	2.56	3.42	2.91	1.89	4.05	

^*∗*^ *P* = 0.05, marginally significantly different.
